# Structural and Functional Studies of Phosphoenolpyruvate Carboxykinase from *Mycobacterium tuberculosis*

**DOI:** 10.1371/journal.pone.0120682

**Published:** 2015-03-23

**Authors:** Iva Machová, Jan Snášel, Jiří Dostál, Jiří Brynda, Jindřich Fanfrlík, Mahavir Singh, Ján Tarábek, Ondřej Vaněk, Lucie Bednárová, Iva Pichová

**Affiliations:** 1 Institute of Organic Chemistry and Biochemistry, Academy of Sciences of the Czech Republic, Prague, Czech Republic; 2 LIONEX diagnostics & Therapeutics, Braunschweig, Germany; 3 Department of Biochemistry, Faculty of Sciences, Charles University in Prague, Prague, Czech Republic; Fundació Institut d’Investigació en Ciències de la Salut Germans Trias i Pujol. Universitat Autònoma de Barcelona. CIBERES, SPAIN

## Abstract

Tuberculosis, the second leading infectious disease killer after HIV, remains a top public health priority. The causative agent of tuberculosis, *Mycobacterium tuberculosis* (Mtb), which can cause both acute and clinically latent infections, reprograms metabolism in response to the host niche. Phosphoenolpyruvate carboxykinase (Pck) is the enzyme at the center of the phosphoenolpyruvate-pyruvate-oxaloacetate node, which is involved in regulating the carbon flow distribution to catabolism, anabolism, or respiration in different states of Mtb infection. Under standard growth conditions, Mtb Pck is associated with gluconeogenesis and catalyzes the metal-dependent formation of phosphoenolpyruvate. In non-replicating Mtb, Pck can catalyze anaplerotic biosynthesis of oxaloacetate. Here, we present insights into the regulation of Mtb Pck activity by divalent cations. Through analysis of the X-ray structure of Pck-GDP and Pck-GDP-Mn^2+^ complexes, mutational analysis of the GDP binding site, and quantum mechanical (QM)-based analysis, we explored the structural determinants of efficient Mtb Pck catalysis. We demonstrate that Mtb Pck requires presence of Mn^2+^ and Mg^2+^ cations for efficient catalysis of gluconeogenic and anaplerotic reactions. The anaplerotic reaction, which preferably functions in reducing conditions that are characteristic for slowed or stopped Mtb replication, is also effectively activated by Fe^2+^ in the presence of Mn^2+^ or Mg^2+^ cations. In contrast, simultaneous presence of Fe^2+^ and Mn^2+^ or Mg^2+^ inhibits the gluconeogenic reaction. These results suggest that inorganic ions can contribute to regulation of central carbon metabolism by influencing the activity of Pck. Furthermore, the X-ray structure determination, biochemical characterization, and QM analysis of Pck mutants confirmed the important role of the Phe triad for proper binding of the GDP-Mn^2+^ complex in the nucleotide binding site and efficient catalysis of the anaplerotic reaction.

## Introduction

Tuberculosis (TB), one of the oldest known human diseases, is still one of the leading infectious disease killers, despite the availability of drugs and the use of attenuated vaccines. The prevalence of multidrug-resistant (MDR) and extensively drug-resistant (XDR) strains of *Mycobacterium tuberculosis* (Mtb), the causative agent of TB, has increased in recent years [1,2]. In addition to the development of Mtb drug resistance, HIV co-infection contributes to the prevalence of TB by dramatically increasing host susceptibility to Mtb. Almost one-third of the world’s population is infected with Mtb. In the majority of these cases, Mtb persists in macrophages in a latent form. Current knowledge of Mtb suggests that adaptation of the bacteria to the host environment is a defining feature of Mtb pathogenicity. Mtb is not subjected to catabolic repression and is able to catabolize multiple carbon sources simultaneously (fatty acids, cholesterol, dextrose, acetate, glycerol, glucose) [3]. This ability likely helps Mtb adapt to the host niche. Numerous studies have shown that central carbon metabolism, which interconnects glycolysis, gluconeogenesis, and the tricarboxylic acid cycle, is altered in latent Mtb. The phosphoenolpyruvate-pyruvate-oxaloacetate node is critical for distribution of carbon flux through central metabolic pathways. The reactions within this node are catalyzed by a set of enzymes that can be regulated under different conditions by various factors, including enzyme activities and specificities, substrate availability, and product inhibition. One of the key enzymes within this node is phosphoenolpyruvate carboxykinase (Pck), which catalyzes the interconversion of phosphoenolpyruvate (PEP) and oxaloacetate (OAA) using ATP or GTP as a cofactor and a divalent cation as an activator. While most bacterial, yeast, trypanosomatic parasite, and plant Pcks are ATP-dependent enzymes, Pcks from human, chicken, rat, and *Mycobacterium smegmatis* require GTP for catalysis [4–10]. The structures of Pck from human, rat, chicken, *Escherichia coli*, and *Trypanosoma cruzi* have been solved [11]. Despite low sequence homology, the ATP- and GTP-dependent Pck families share a conserved active site architecture. In GTP-dependent Pcks, Mn^2+^ preferably associates with the free enzyme, and an additional metal, usually Mg^2+^, is bound with the nucleotide [12–15]. Structural studies of GTP-dependent Pcks [11] indicated the presence of C- and N-terminal lobes, with the active site between the two lobes. The C-terminal lobe contains the nucleotide binding domain and Pck-specific domain. Structural and mutagenesis studies have confirmed the importance of three mobile loops in the structure. The R- and P-loops are involved directly in substrate binding and catalysis, and the flexible Ω-loop lid domain, which undergoes an open-closed transition during substrate binding, stabilizes the correct position of the substrate for catalysis [8,9,12] and protects the enolate intermediate from protonation.

Mtb Pck is a GTP-dependent enzyme that under standard conditions catalyzes the gluconeogenic reaction leading to production of PEP. However, under reducing conditions, it favors the anaplerotic OAA synthesis [16]. The role of Pck in fixation of CO_2_ during OAA biosynthesis has also been reported for slowly or intracellularly growing Mtb [17,18] and Mtb cultivated in conditions mimicking hypoxia arrest [19]. Reversible flux through the PEP-OAA node enables Mtb to tune the balance of catabolic reactions and replenish the tricarboxylic acid cycle products. Marrero et al. [20] showed the importance of Pck for Mtb survival in macrophages in a murine model of TB and suggested an essential role for Mtb Pck for gluconeogenesis. Mtb Pck represents a potential target for drug design due to its role in persistent Mtb. Solution of the three-dimensional structure of human Pck revealed the presence of a unique binding site for GTP, which has opened possibilities for development of specific compounds targeted against this structure motif [9].

Here, we present the crystal structures of Pck-GDP and Pck-GDP-Mn^2+^ complexes and an analysis of the contributions of key interacting residues in the GTP/GDP binding site to the enzyme activity using biochemical and quantum mechanical analyses of Pck mutants. Our findings suggest that changes in cellular cation concentrations contribute to Pck catalysis and function in various stages of Mtb infection.

## Materials and Methods

### Cloning, expression, and purification of Mtb Pck

Cloning, expression, and purification of Mtb Pck has been described [16]. Briefly, Pck (Rv0211) containing an N-terminal His-tag was expressed in *E*. *coli* BL21(DE3). Harvested cells were lysed by multiple freeze-thaw cycles and addition of lysozyme. The cell supernatant was loaded onto Talon chromatography resin (Clontech), and the column was washed with 20 mM Tris-HCl, pH 7.4, containing 500 mM NaCl and 10 mM imidazole. Pck was eluted by sequentially increasing the imidazole concentration (50, 150, 300, 500, and 800 mM) in the elution buffer. Pck-containing fractions were dialyzed against 50 mM Tris-HCl, pH 7.4, 300 mM NaCl, 5 mM 2-mercaptoethanol; concentrated; and purified on FPLC HiLoad 16/60 (Superdex 75 pg, GE Healthcare) equilibrated with 20 mM Tris-HCl, pH 8, 500 mM NaCl. Traces of metal ions were removed using Chelex resin (BioRad) in Pck samles prepared for measurements of activation of Pck by different metal ions or for crystallization experiments. 100 ml of Pck solution was gently shaken with 5 g of Chelex resin for 1 hour at 4°C. The Chelex resin was removed by filtration.

### Site-directed mutagenesis

Site-directed mutagenesis was performed according to the QuikChange protocol (Stratagene) with minor modifications. The complementary primers (1 μg/ml) (Phe502: forward: 5'-CTTCGTCAACTGG**GCC**CGTCGCGGTGACG, reverse: 5'-CGTCACCGCGACG**GGC**CCAGTTGACGAAG; Phe510: forward: 5'-GGTGACGACGGTCGC**GCC**CTGTGGCCGGGCTTCG, reverse: 5'-CGAAGCCCGGCCACAG**GGC**GCGACCGTCGTCACC; Phe515: forward: 5'- CCTGTGGCCGGGC**GCC**GGCGAGAACAGCCGG, reverse: 5'-CCGGCTGTTCTCGCC**GGC**GCCCGGCCACAGG) were mixed in *Pfu* polymerase reaction buffer with 0.2 mM dNTPs and 50 ng plasmid template containing the Pck coding sequence. Upon addition of *Pfu* polymerase (2 U/reaction), the cycling reaction (linear amplification reaction) was started. The following program was used: denaturation at 94 ^°^C (50 sec), annealing at 55 ^°^C (50 sec), and extension at 72 ^°^C (8 min) with 19 repeats. Following *Dpn*I restriction digest (37 ^°^C, 12 h) to remove the template plasmid, the mixture was transformed into DH5α ultracompetent cells. Plasmids were purified from single colonies and sequenced.

### Enzyme activity measurement

Pck activity was determined as previously described [16]. To monitor the rate of the anaplerotic reaction (OAA formation), the Pck-catalyzed reaction was coupled with the subsequent reaction catalyzed by malatedehydrogenase from *Thermus flavus* (Sigma Aldrich). The reaction progress was followed by monitoring the decrease in absorbance at 340 nm due to NADH oxidation to NAD^+^. The standard reaction mixture contained 100 mM HEPES-NaOH, pH 7.2, 100 mM KHCO_3_, 37 mM DTT, 2 mM PEP, 1 mM GDP, 2 mM MgCl_2_, 0.1 mM MnCl_2_, 2 U/ml MDH, and 0.25 mM NADH. Each reaction was started by addition of the essential divalent cations (Mg^2+^ and Mn^2+^). Measurements of Pck activation by different metal ions were carried out in the same mixture but without Mn^2+^ and Mg^2+^, and reactions were started by addition of the tested ions. To monitor the gluconeogenic reaction (PEP formation), the Pck-catalyzed reaction was coupled with reactions catalyzed by pyruvate kinase (PK, Roche) and lactate dehydrogenase (LDH, Roche). In this set-up, the reaction rate corresponds to the decrease in absorbance at 340 nm due to oxidation of NADH to NAD^+^. The typical reaction mixture was composed of 100 mM HEPES-NaOH, pH 7.2, 0.3 mM OAA, 0.2 mM GTP, 2 mM MgCl_2_, 0.2 mM MnCl_2_, 10 mM DTT, 10 U/ml LDH, 3 U/ml PK, and 0.2 mM NADH [16]. For measurements of Pck activation by different metal ions, we used the same reaction mixture free of Mn^2+^ and Mg^2+^. The kinetic data were fitted using a nonlinear least squares regression analysis (SigmaPlot 11.0).

### Analytical ultracentrifugation

Sedimentation analysis was performed using a ProteomeLabXL-I analytical ultracentrifuge equipped with an An50Ti rotor (BeckmanCoulter). Pck (0.39 mg/ml) was dialyzed against 50 mM HEPES-NaOH, pH 7.4, 2 mM MgCl_2_, 0.1 mM MnCl_2_, 50 mM KCl, 10 mM 2-mercaptoethanol. The same buffer was also used as a reference and sample dilution buffer. Sedimentation velocity experiments were carried out at 48,000 rpm and 20°C; 200 absorbance scans were recorded at 280 nm at 3 min intervals with 30 μm spatial resolution. Buffer density and Pck partial specific volume were estimated with SEDNTERP 1.09 (www.jphilo.mailway.com). Data were analyzed with SEDFIT 13.0b [21]. The sedimentation equilibrium experiment was performed with a Pck concentration of 0.13 mg/ml at 10–12–14–16–18–20–22,000 rpm at 4°C. Absorbance data were collected at 280 nm by averaging 20 scans with 10 μm spatial resolution after 30 h (first scan) or 18 h (consecutive scans) of achieving equilibrium and were globally analyzed with SEDPHAT 10.54e [22] using a non-interacting discrete species model.

### Analytical gel-permeation chromatography

The protein sample was dialyzed against buffer (50 mM Tris-HCl, pH 7.4, 100 mM NaCl, 5 mM 2-mercaptoethanol) and concentrated to 13 mg/ml using an Amicon Ultra centrifugal filter unit (Millipore). A portion of the sample was equilibrated with 2 mM MgCl_2_, 0.1 mM MnCl_2,_ and 1 mM GDP or 2 mM MgCl_2_, 0.1 mM MnCl_2_, 2 mM PEP, and 1 mM GDP. Individual mixtures were loaded onto an FPLC gel filtration column (Superdex 200 10/300 GL) alongside protein standards with known molecular masses. The protein content of collected fractions was determined by measuring the absorbance at 280 nm.

### Isothermal titration calorimetry

Isothermal titration calorimetric (ITC) measurements were carried out at 25 ^°^C using a VP-ITC system (MicroCal, GE Healthcare Life Sciences). Typically, 10 μl aliquots of 220 μM GDP or GTP were injected stepwise into a sample cell containing 1.43 ml of a 8.5 μM Pck protein solution until saturation was achieved. Control dilution experiments in which GDP or GTP was injected into buffer alone were also performed. The exact concentration of Pck was determined by HPLC amino acid analysis. The binding constants were determined with MicroCal software implemented in Origin 7.0 (MicroCal, GE Healthcare Life Sciences).

### Protein crystallization

Two different complexes were crystallized: 1) Pck-GDP and 2) Pck-GDP-Mn^2+^. Mtb Pck was pre-incubated either with 1 mM GDP or with 1 mM GDP and 0.1 mM Mn^2+^ for 1 h on ice, then concentrated to 13 mg/ml. Initial crystallization trials were performed with the help of a Gryphon crystallization workstation (Art Robbins Instruments) by the sitting drop vapor diffusion method at 19°C in 96-well plates; 0.2 μl protein solution was mixed with 0.2 μl well solution, and the mixture was equilibrated over 200 μl reservoir solution. The PEGs Suite I, PEGs Suite II, and JSCG Core I Suite (QIAGEN) were used for the crystallization condition screen. Initial microcrystals appeared in several days under the following conditions: 0.1 M sodium acetate, pH 4.6, containing 30% PEG 300 or 40% PEG 200. Further optimization involved changing to the hanging drop mode in 24-well crystallization plates (EasyXtal DG-Tool, QIAGEN). Final crystals were obtained by mixing 3 μl Mtb Pck-GDP or Mtb Pck-GDP-Mn^2+^complex with 1 μl reservoir solution composed of 0.1 M sodium acetate, pH 4.6, 30% PEG 300. Crystals were directly cryocooled in liquid nitrogen.

### Data collection and structure determination

Diffraction data for complex Pck-GDP- Mn^2+^ were collected to 1.8 Å resolution at 100 K using the MX14.2 beamline at BESSY, Berlin, Germany. Diffraction data for crystal without Mn^2+^ ion were collected to 2.6 Å resolution at 120 K using an in-house diffractometer (Nonius FR 591) connected to 345 mm MarResearch image plate detector. The diffraction data of both data sets were integrated and reduced using XDS [23] and its graphical interface XDSAPP [24]. The structures of both complexes were solved by molecular replacement using the program Molrep. The search model was derived from the structure of rat Pck-GDP complex (PDB code 3DTB). Model refinement was carried out using the program REFMAC 5.2 from the CCP4 package. Manual building was performed using Coot. The quality of the final model was validated with Molprobity. Crystal parameters, data collection and refinement statistics are summarized in Table 1. Fig. 4 and S1 Fig. showing structural representations were prepared with the program PyMOL. Atomic coordinates and experimental structure factors have been deposited in the Protein Data Bank under code 4R43 (Mtb Pck-GDP-Mn^2+^) and 4RCG (Mtb Pck-GDP).

**Table 1:** Data collection statistics.

X-ray structure	Mtb Pck-GDP Mn2+	Mtb Pck-GDP
PDB code	4R43	4RCG
**Data collection statistics**		
Space group	*C 2 2 2*_*1*_	*C 2 2 2*_*1*_
Cell parameters (Å, °)	α = β = γ = 90	α = β = γ = 90
Number of molecules in AU	1	1
Wavelength (Å)	0.979402	1.541790
Resolution (Å)	50–1.80 (1.90–1.80)	50–2.60 (2.76–2.60)
Number of unique reflections	72276	24338
Multiplicity	4.4	6.0
Completeness (%)	98.8 (96.2)	99.0(95.1)
R_meas_^a^	5.4 (76.3)	13.0(55.0)
Average *I*/σ(*I*)	16.1 (2.96)	10.6(3.1)
Wilson B (Å^2^)^b^	33.3	37.7
**Refinement statistics**		
Resolution range (Å)	47.55–1.80 (1.85–1.80)	48.80–2.60 (2.67–2.60)
No. of reflections in working set	68,999 (4786)	23,134 (1441)
No. of reflections in test set	3,632 (253)	1,218 (75)
R value (%)^c^	20.7 (49.2)	21.0 (31.0)
R_free_ value (%)^d^	24.3 (53.2)	27.0 (40.0)
RMSD bond length (Å)	0.018	0.015
RMSD angle (°)	1.95	1.89
Number of atoms in AU	4,816	4,601
Number of protein residues in AU	601	598
Number of solvent atoms in AU	98	21
Mean B value (Å^2^)	36.1	36.8
**Ramachandran plot statistics** ^**e**^:		
Residues in favored regions (%)	95.7	94.05
Residues in allowed regions (%)	3.98	5.44

Rmerge = ΣhklΣi*I*i(hkl)—⟨*I*(hkl)⟩|/ ΣhklΣ i *I*i(hkl), where *I*i(hkl) is the individual intensity of the ith observation of reflection hkl and ⟨*I*(hkl)⟩ is the average intensity of reflection hkl with summation over all data.

^b^ Wilson B calculated with the Sfcheck program from the CCP4 suite (CCP4, 1994).

^c^ R-value = ||*F*_o_|—|*F*_c_||/|*F*_o_|, where *F*_o_ and *F*_c_ are the observed and calculated structure factors, respectively.

^d^ R_free_ is equivalent to R value but is calculated for 5% of the reflections chosen at random and omitted from the refinement process [25].

^e^ as determined by MolProbity [26].

### Molecular modeling

To study interactions of GDP with phenylalanine residues in the Mtb Pck active site computationally, we used both the binary complex of Mtb Pck-GDP (the PDB code 4RCG) and the ternary complex of Mtb Pck-GDP-Mn^2+^ (the PDB code 4R43). Hydrogens were added with the Reduce and LeaP modules in the AMBER package [27]. Protonation of histidines was assigned individually on the basis of visual inspection of their surroundings. The protein N-terminus, lysines, and arginines were positively charged, whereas the C-terminus, GDP (charge of -3), glutamates, and aspartates were negatively charged. The added atoms were relaxed by annealing (10 ps) from 600 to 10 K and gradient optimization in the SANDER module of the AMBER 11 package [27].

For calculations, we utilized a hybrid QM/MM and QM/SQM methodologies. The coupling between the high level part (described by quantum mechanics (QM)) and low level part (described by semiempirical QM (SQM) or molecular mechanics (MM)) was performed with an in-house program (CUBY3) using a subtractive scheme of an ONIOM type [28] and a mechanical embedding. It calls Turbomole [29] for QM, MOPAC [30] for SQM and AMBER [27] for MM. The QM part was treated with DFT-D/B-LYP/SVP [31] for optimization and DFT-D3/TPSS/TZVPP [32] for single-point energy calculations. The QM region comprises GDP and residues Ala273, Cys274, Gly275, Lys276, Thr277, Trp501, Phe502, Phe510, and Phe515. In the case of the ternary Mtb Pck-GDP-Mn^2+^ complex, the QM region also includes Mn^2+^ and four water molecules (i.e. the first solvation shell). The SQM part was treated according to the PM6-D3H4 method with the linear scaling procedure MOZYME [33,34]. The MOZYME procedure enables us to calculate extended biomolecular complexes. However, only closed shell calculations are allowed in this procedure. Ions that are definitely open shell, such as Mn^2+^, cannot be run. Consequently, the QM/SQM method could only be applied to the binary Mtb Pck-GDP complex. The protein surroundings were modeled using the COSMO implicit solvent model [35] in the QM/SQM method.

We used the parm03 force field [36] of the Amber family of force fields for protein in the MM part. The MM parameters for Mn^2+^ were adopted from previous study [37]. The atoms of the ligand were described by the General Amber Force Field (GAFF) [36]. The partial charges on the ligand atoms were assigned according to the Restricted Electrostatic Potential (RESP) procedure calculated on the HF/6–31G level [38] to remain consistent with the description of the protein. The protein surroundings were modeled using the Generalized Born solvent model in the QM/MM method.

Residues farther than 10 Å from GDP were frozen during gradient optimization. For optimization by the QM/SQM method, only residues within 12 Å of GDP were considered.

The contributions of Phe residues in the active site to GDP binding were examined by virtual alanine scanning [39,40]. The energy contributions of individual Phe residues to the GDP binding (ΔΔG'_int_) were calculated as the difference between the original GDP binding energy (ΔG'_int_) at the QM/SQM level with the wild type amino acid and the new ΔG'_int_ with the mutated alanine residue.

The evaluation of ΔG'_int_ of the Mtb Pck-GDP-Mn^2+^ complex requires calculation of the Mtb Pck-Mn^2+^ complex without GDP. In these calculation, an additional single explicit water molecule (i.e. the first solvation shell) was used to screen the Mn^2+^ charge and decrease the error of the ΔG'_int_.

### Electron paramagnetic resonance spectroscopy

Electron paramagnetic resonance (EPR) spectra were recorded on an EMX^plus^-10/12 CW (continuous wave) spectrometer (Bruker, Germany) equipped with a *Premium X*-band microwave bridge. All measurements were carried out within a double rectangular resonator (ER410500DR, Bruker, Germany). The enzyme (17 μM) used for EPR measurements was first incubated in buffer (50 mM MOPS, pH 7.4, 50 mM KCl) and then passed through Chelex-100 resin to remove any trace metal ions. The concentration of Mn^2+^ was varied from 0 to 1 mM. Free Mn^2+^ present in each Pck sample was measured after sampling into 1 mm (i.d.) glass capillaries (Brand micropipettes, Germany) with a calibrated volume of 50 μl. Samples were placed into the front and rear cavities of the ER410500DR. Each EPR spectrum was recorded as an accumulation of several sweeps in order to enhance the signal/noise ratio, with the following parameters: sweep width = 100 mT, power = 7.96 mW, modulation amplitude = 0.24 mT, time constant = 10.24 ms, conversion time = 16 ms, gain = 10^5^, resolution = 0.025 mT (4001 points). The g-factor was determined by the built-in spectrometer frequency counter and the external ER036TM NMR-Teslameter (both Bruker, Germany). The dissociation constant was calculated by curve fitting to equation (1), where [Mn^2+^] is the concentration of free Mn^2+^, ʋ is the number of moles of Mn^2+^ bound per enzyme monomer, K_D_ is the equilibrium dissociation constant, and m is the stoichiometry of binding.

### Circular dichroism

Circular dichroism (CD) spectra were collected on Jasco-815 spectrometer in spectral range 180 nm—300 nm at room temperature. The standard 0.1cm quartz cell (Hellma) was used with the following experimental setup: 2 scans, 0.5 nm steps, 10 nm/min speed, 16 s time constant, 1 nm spectral bandwidth, sample concentration of the samples diluted at 20 mM Tris-HCl, pH 7.4, were 1.3 μM (wt), 1.3 μM (F502A), 1.5 μM (F510A) 1.4μM (F515A), 1.5μM (F502, 515A), 1.8 μM (F510, 515A), 0.6 μM (F502, 510A), 1.75 μM (F502, 510, 515A). After baseline correction, the final spectra were expressed as a molar ellipticity θ (deg.cm^2^.dmol^−1^) per residue. The numerical analysis of the secondary structure and secondary-structure assignment was performed using an online Circular Dichroism Analysis program Dichroweb (http://dichroweb.cryst.bbk.ac.uk) [41].

## Results

Although the key role of Pck in regulating the central carbon metabolism of Mtb has been confirmed, little is known about the properties of this enzyme. Recently, we described how Mtb Pck specificity is regulated by reducing conditions and interaction with proteins involved in antioxidant defense and maintenance of a reduced intracellular state [16]. However, a more detailed characterization of this enzyme, which has been predicted to be a potential target for drug design, was lacking.

### Mtb Pck is a monomeric enzyme

**Fig 1:**
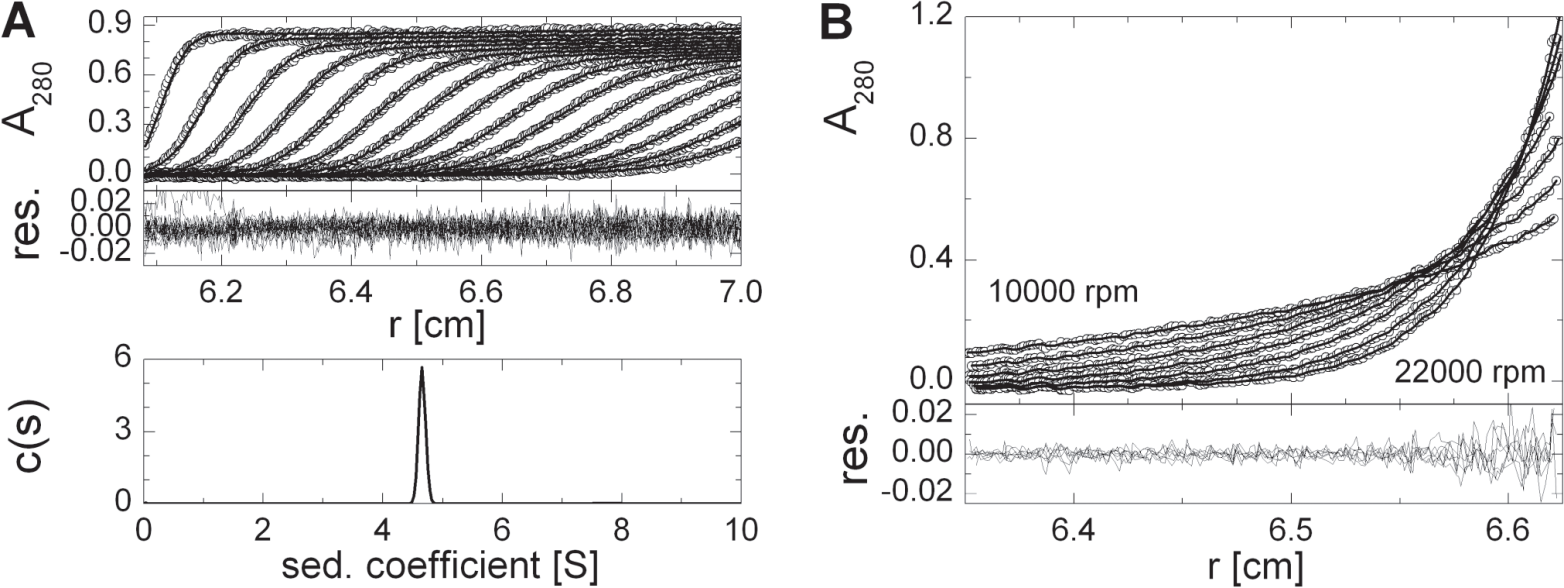
Determination of the oligomeric state of Mtb Pck by analytical ultracentrifugation. (A) Sedimentation velocity experiment. Fitted data (upper panel) with residual plots (middle panel) representing the accuracy of the fit are shown together with the calculated continuous size distribution(s) of the sedimenting species (lower panel). (B) Equilibrium sedimentation distribution of Pck at 10–12–14–16–18–20–22,000 rpm. The upper panel shows absorbance data with fitted curves (non-interacting discrete species model, lines); the lower panel shows residuals derived from the fitted data.

To analyze the oligomeric state of Mtb Pck, we used analytical gel permeation chromatography and analytical ultracentrifugation (sedimentation velocity and sedimentation equilibrium experiments). Mtb Pck (13 mg/ml) solubilized in HEPES-NaOH buffer pH 7.4 containing 2 mM MgCl_2_, 0.1 mM MnCl_2_ was loaded onto a Superdex 200 10/300 GL analytical column alongside molecular mass standards. The major peak eluted at the position corresponding to the estimated molecular mass of a monomer (68.7 kDa). Analytical ultracentifugation experiments also indicated that Pck exists as a monomer, with sedimentation coefficient s_20,w_ 4.89 S° (Fig. 1A). The moderately elongated particle, with approximate dimensions of 3.5–4.5 × 8.5–11.5 nm, corresponds to a mass lower than that expected for any potential oligomers. Sedimentation equilibrium experiments (Fig. 1B) enabled us to calculate an average molecular mass of 67 kDa for one ideal particle, which correlates with the expected mass of the Pck monomer (69.4 kDa) within the limits of method precision. Finally, we tested whether the oligomeric state of recombinant Pck changes in the presence of 2 mM PEP or 1 mM GDP. Both sedimentation velocity and equilibrium experiments were performed, but no evidence of protein oligomerization upon addition of PEP or GDP was detectable.

### Divalent metal cations have different effects on the gluconeogenic and anaplerotic reactions catalyzed by Mtb Pck

Kinetic studies of activation of Pcks from several sources indicated diverse dependence on metal cations. Mn^2+^ has been shown to be the best activator for cytosolic Pcks from chicken, guinea pig, and human [9,14,42]. Mitochondrial Pck from guinea pig displays better activity with Mg^2+^ [42]. A synergic effect of Mg^2+^ and Mn^2+^ was observed for mitochondrial Pcks from rat and chicken liver [43,44].

**Fig 2:**
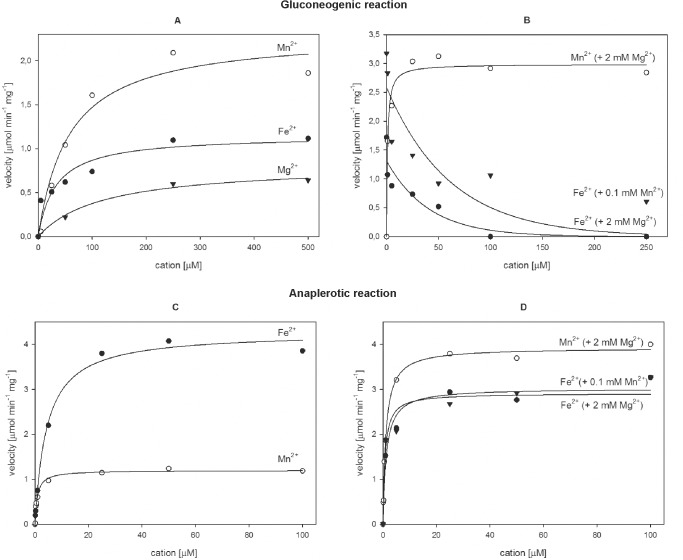
Influence of Mn^2+^, Fe^2+^, and Mg^2+^ cations on Pck activity. Activation of the gluconeogenic reaction by (A) Mn^2+^, Fe^2+^, or Mg^2+^ alone (B) Mn^2+^ in the presence of 2 mM Mg^2+^, and Fe^2+^ in the presence of 0.1 mM Mn^2^ and 2mM Mg^2^. Activation of the anaplerotic reaction by (C) Mn^2+^, Fe^2+^, or Mg^2+^ alone and by (D) Mn^2+^ in the presence of 2 mM Mg^2+^, and Fe^2+^ in the presence of 0.1 mM Mn^2^ and 2 mM Mg^2^.

To investigate the dependencies of the gluconeogenic and anaplerotic reactions catalyzed by Pck on metal cations present in Mtb, we collected kinetic data for Mtb Pck in the presence of Mn^2+^, Fe^2+^, and Mg^2+^ alone or in combination. Trace metal ions present in purified Pck samples were removed by incubation with Chelex resin before each experiment. The gluconeogenic reaction was only moderately stimulated in the presence of Mn^2+^, Mg^2+^, and Fe^2+^ alone (Fig. 2A, Table 2). When Mn^2+^ or Fe^2+^ was present together with 2 mM Mg^2+^, the metal ion dependency curves completely changed (Fig. 2B). We observed a two orders of magnitude increase in Pck catalytic efficiency in the presence of both Mn^2+^ and Mg^2+^ compared to Mn^2+^ alone. However, the gluconeogenic reaction was inhibited by increasing concentrations of Fe^2+^ in the presence of 2 mM Mg^2+^ or 100 μM Mn^2+^ (Fig. 2B).

**Table 2:** Kinetic constants for wt Mtb Pck in the presence of different divalent cations.

**Gluconeogenic reaction** ^***a***^			
**Variable cations**	**V**_**max**_	**K**_**m**_	**V**_**max**_/**K**_**m**_
**(concentration range tested)**	**(**μ**mol·min** ^**−1**^ **·mg** ^**−1**^)	**(**μ**M)**	
Mn^2+^ (0–500 μM) single cation	2.31 ± 0.20	57.45 ± 16.40	0.04
Mg^2+^ (0–500 μM) single cation	0.82 ± 0.08	119.13 ± 36.86	0.006
Fe^2+^ (0–500 μM) single cation	1.15 ± 0.13	32.58 ± 14.70	0.04
Mn^2+^ (0–250 μM) + 2 mM Mg^2+^	2.81 ± 0.16	0.72 ± 0.38	3.89
Fe^2+^ (0–250 μM) + 2 mM Mg^2+^	n.d.	n.d.	n.d.
Fe^2+^ (0–250 μM) + 100 μM Mn^2+^	n.d.	n.d.	n.d.
**Anaplerotic reaction** ^***b***^			
**Variable cations**	**V**_**max**_	**K**_**m**_	**V**_**max**_/**K**_**m**_
**(concentration range tested)**	**(**μ**mol·min** ^**−1**^ **·mg** ^**−1**^)	**(**μ**M)**	
Mn^2+^ (0–100 μM) single cation	1.20 ± 0.04	0.87 ± 0.16	1.38
Mg^2+^ (0–100 μM) single cation	n.d.	n.d.	n.d.
Fe^2+^ (0–100 μM) single cation	4.27 ± 0.11	4.40 ± 0.52	0.97
Mn^2+^ (0–100 μM) + 2 mM Mg^2+^	3.92 ± 0.07	1.08 ± 0.10	3.63
Fe^2+^ (0–100 μM) + 2 mM Mg^2+^	3.02 ± 0.15	1.24 ± 0.39	2.44
Fe^2+^ (0–100 μM) + 100 μM Mn^2+^	3.17 ± 0.12	2.89 ± 0.77	1.10

^*a*^For gluconeogenic reaction 100 mM HEPES-NaOH, pH 7.2, 0.3 mM OAA, 0.2 mM GTP, 2 mM MgCl_2_, 0.2 mM MnCl_2_, 10 mM DTT, 10 U/ml LDH, 3 U/ml PK and 0.2 mM NADH was used.

^*b*^For anaplerotic reaction 100 mM HEPES-NaOH, pH 7.2, 100 mM KHCO_3_, 37 mM DTT, 2 mM PEP, 1 mM GDP, 2 mM MgCl_2_, 0.1 mM MnCl_2_, 2 U/ml MDH and 0.25 mM NADH was used.

n.d.: Kinetic constants cannot be calculated.

Manganese and iron alone displayed stimulatory effects on anaplerotic reaction catalyzed by Mtb Pck in contrast to magnesium that did not activate this reaction (Fig. 2C, Table 2). The highest catalytic efficiency for Pck anaplerotic reaction was detected in the presence of Mn^2+^ with Mg^2+^. In contrast to gluconeogenic reaction, we observed increased activation of anaplerotic reaction by Fe^2+^ with Mg^2+^ and Fe^2+^ with Mn^2+^ cations (Fig. 2D, Table 2). Activation of anaplerotic reaction by all tested cations required reducing conditions. These results indicate that Fe^2+^ can regulate the Mtb Pck catalysis. The increasing concentration of iron in the presence of commonly occurring Mn^2+^ and Mg^2+^ in Mtb efficiently activates the anaplerotic reaction and inhibit gluconeogenic formation of phosphoenolpyruvate.

### X-ray structure of Mtb Pck

**Fig 3:**
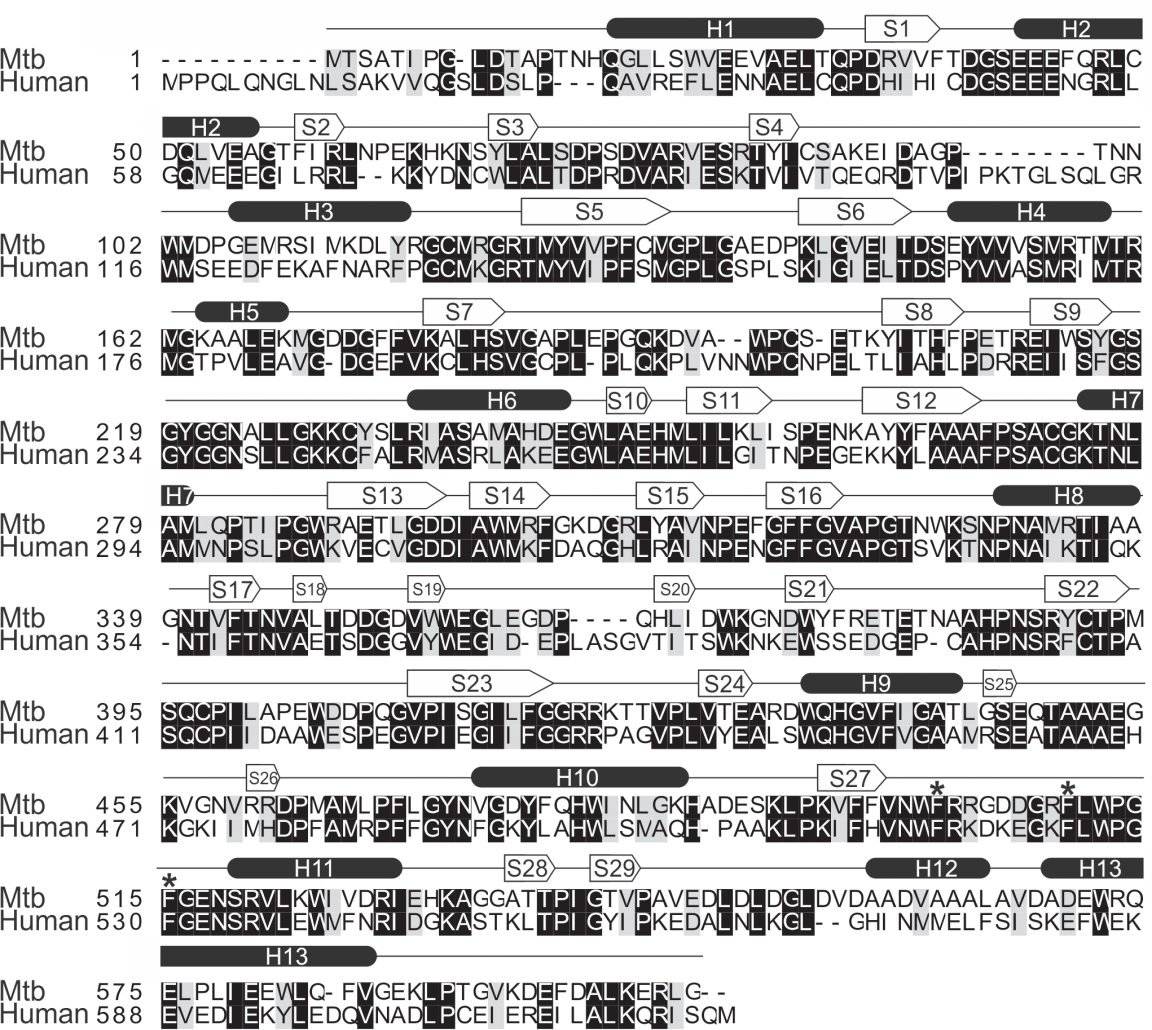
Sequence alignment of Mtb and human cytosolic Pcks. The alignment was created with Clustal W2. The UniProt accession numbers of the sequences are P9WIH3 for Mtb Pck and P35558 for human Pck. Black and grey backgrounds denote identical and similar amino acid residues, respectively. Secondary structure elements found in Mtb Pck are shown as ellipses (helices) and arrows (strands). The aromatic residues Phe501, Phe509, and Phe514 are indicated by asterisks.

The primary structures of Mtb and human Pck share 50% identity and 66% similarity (Fig. 3). To explore the Mtb GTP binding pocket as a potential site for Pck inhibition, we determined the crystal structures of Mtb Pck in complex with GDP and Mn^2+^ and with GDP. The Pck-GDP-Mn^2+^ and Pck-GDP complexes crystallized in the *C 222*_*1*_ space group with one protein molecule in the asymmetric unit. The crystal structures of both complexes were determined by molecular replacement using the structure of rat Pck (PDB code 3DTB) as a search model. The final dataset was collected to 1.8 Å resolution for Pck-GDP-Mn^2+^ and to 2.6 Å resolution for Pck-GDP complex, respectively. The protein residues of both complexes could be modeled into a well-defined electron density map, except the 6 N-terminal residues connected with the His-tag that form highly flexible and disordered region. Additional disordered region is present between residues Ala448-Asn458, forming flexible loop in the Pck-GDP complex. Data collection statistics and refinement statistics are summarized in Table 1. Structures of Pck-GDP-Mn^2+^ and Pck-GDP complexes are very similar, the Cα root-mean-square deviation (RMSD) is 0.363 Å. The differences bigger than 1Å are located within the N-terminal residues Gly8—Thr11 and in residues Arg81, Gly190, and Glu259. These residues are located within various flexible loops.

**Fig 4:**
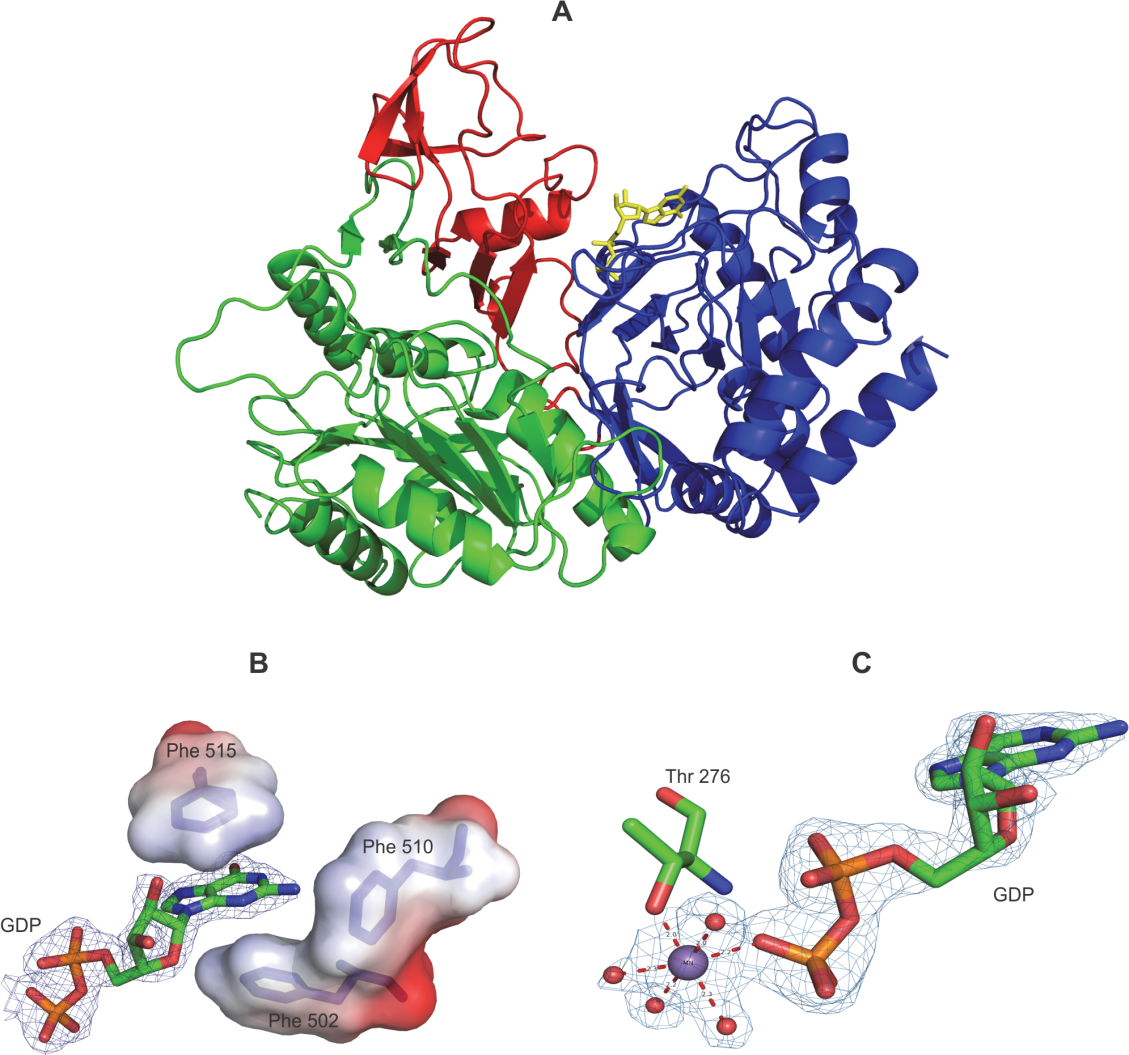
The crystal structure of Mtb Pck. (A) Overall three-dimensional structure and secondary structural elements of Pck-GDP-Mn^2+^ complex. The protein is shown in cartoon representation. The N-terminal domain (residues 1–241), C-terminal domain (residues 242–309 and 407–604) are colored green and blue, respectively), and small N-terminal subdomain (residues 310–406) is red. GDP is shown as yellow sticks. (B) Detail of the guanine base of GDP, which interacts in the Pck active site with aromatic residues Phe502, Phe510, and Phe515. The GDP F_o_—F_c_ electron density map rendered at 3σ prior to the inclusion of the ligands into the model is shown as a blue mesh. The carbon atoms of GDP are shown in green, oxygen and nitrogen are colored red and blue, respectively. Phosphate is orange. The residues Phe502, Phe510 and Phe515 are depicted in blue sticks and their solvent accessible surface are colored by electrostatic potential (red for negative, blue for positive). (C) Interactions of Mn^2+^ (purple sphere) in the active site of Pck-GDP-Mn^2+^ complex. Mn^2+^ is octahedrally coordinated by the side chains oxygen of Thr276, O3B atom of GDP and four water molecules (shown as red spheres). The 2F_o_—F_c_ electron density map rendered at 1.5σ p is shown as blue mesh. Coordinate bonds are shown as dashed lines. The carbon atoms of GDP and Thr276 are shown in green, oxygen, nitrogen and phosphate are colored red, blue and orange, respectively.

The overall fold of both complexes is very similar to that of human and rat Pck (PDB codes 1KHB, 3DTB), with RMSD of 0.981 Å and 0.740 Å, respectively. Like human cytosolic Pck, Mtb Pck consists of two domains (Fig. 4A): the N-terminal domain containing 337 residues (residues Ile1 to Glu241 and small subdomain comprising residues Val310 to Gln406) and a slightly smaller C-terminal or mononucleotide-binding domain of 264 residues (residues Gly242 to Ala309 and Gly407 to Gly604). The Mtb Pck structure contains typical Pck motifs, including mobile loops R (residues Val79 to Thr86), P (residues Phe269 to Asn277), and Ω (residues Gln448 toAsn458). The structure also contains the conserved reactive cysteine residue (C273) and Phe residues (F502, F510, and F515) within the GTP binding site. The P-loop (residues Phe269 to Asn277) is located within the C-terminal domain and contains the hyperactive Cys273 [16]. Connections between the N-terminal and C-terminal domains are formed by central α-helix H6 and by two polypeptide connections extending from β-strands S16 and S22 of the N-terminal domain to β-strands S10 and S15 of the C-terminal domain (Figs. 3 and 4). No disulfide bridges are present in the structure of Mtb Pck.

The identical interactions were found for GDP in the nucleotide binding sites of both complexes. Alignment of positioning of Phe residues in Pck-GDP and PCK-GDP-Mn^2+^ nucleotide binding sites is illustrated in S1 Fig. Electrostatic interactions and hydrogen bonds are formed between residues Ala272, Gly274, Lys275, Thr276, and Asn277 from the P loop and the phosphoryl oxygen atoms of GDP (Table 3) and contribute to the stabilization and correct orientation of GDP in the active site. The interactions between Mtb Pck and the phosphoryl oxygen atoms are similar to those found in rat, human, and chicken Pck [8,9,12]. Numerous interactions contributing to stabilization of GDP in complex with Pck are formed by binding pocket residues that interact with the guanine base and ribose sugar of GDP. Hydrogen bonds are formed between the guanine base and residues Phe515 and Asn518; additional bonds are present between Arg420 and Ala272 and the ribose sugar. Furthermore, the aromatic residues Phe502, Phe510, and Phe515 form a pocket with three walls and strongly contribute to GDP binding by π-stacking interactions with the guanine base sandwiched between the side chains of Phe502 and Phe515 (Fig. 4B and Table 3). Another stabilizing factor is the π-interaction of the Phe510 side chain with the N-2 of the guanine ring.

**Table 3:** Mtb Pck Amino acid (AA) residues forming hydrogen bonds and close contacts (˂ 3.7Å) with guanosine-5'-diphosphate (GDP) in Mtb Pck-GDP-Mn^2+^ complex.

Mtb Pck-GDP-Mn^2+^								
Guanine base			Ribose sugar			Phosphoryl oxygen atoms		
AA atom	GDP	Distance	AA atom	GDP	Distance	AA atom	GDP	Distance
	atom	(Å)		atom	(Å)		atom	(Å)
Asn 277 ND2	C8	3.50	Ala 272 O	C4´	3.67	Ala 272 N	O2B	3.49
Trp 501 CD1	O6	3.44	Ala 272 C	C5´	3.53	**Ala 272 N**	**O3B**	**2.8**
Phe 502 CE1	N3	3.38	Ala 272 O	C5´	3.44	Gly 274 CA	O1A	3.33
Phe 502 CE1	C2	3.60	**Arg 420 NH2**	**O4´**	**2.92**	Gly 274 C	O1A	3.41
Phe 510 CZ	N2	3.55	Arg 420 NH2	C4´	3.49	**Gly 274 N**	**O3A**	**2.90**
Phe 510 CE2	N2	3.46	**Arg 420 NH2**	**O4'**	**2.95**	Gly 274 CA	O3A	3.16
**Phe 515 N**	**O6**	**2.92**	Phe 515 CZ	O2´	3.5	Gly 274 N	O2B	3.21
Phe 515 N	C6	3.66				**Lys 275 N**	**O2B**	**2.86**
Phe 515 CE2	C2	3.70				Lys 275 N	O3A	3.36
Phe 515 CZ	N3	3.55				**Thr 276 OG1**	**O1B**	**3.00**
Phe 515 CZ	C4	3.39				Thr 276 N	O1A	3.40
**Asn 518 ND2**	**O6**	**2.91**				**Thr 276 N**	**O1B**	**2.90**
						Thr 276 CB	O1B	3.36
						**Asn 277 N**	**O1A**	**2.97**
						Asn 277 CB	O1A	3.57
						**Asn 277 ND2**	**O1A**	**2.87**

Two binding sites for Mn^2+^, denoted as metal binding sites 1 and 2, have been identified in Pck from *E*. *coli*, human, *T*. *cruzi*, and *Anaerobiospirillium succiniciproducens* [9,45–48]. The structural alignment of rat, human, and Mtb Pck predicted that Mtb Pck metal binding site 1 would be formed by residues Asp295, Lys249, and His249 and metal binding site 2 by residues Thr276 and Asp295. However, only one tightly bound Mn^2+^ ion was found in our model based on the coordination geometry and refined B factors in the presence of 0.1 mM Mn^2+^ in the crystallization solution. The Mn^2+^ coordination sphere is formed by four water molecules, oxygen from the GDP phosphoryl group (O3B), and OG1 oxygen atoms of Thr276 (Fig. 4C). These hydrogen bonds, together with an additional water molecule, form the octahedral environment of Mn^2+^ in metal binding site 2 of Mtb Pck. In human Pck, Mn^2+^ was captured in metal binding site 1 [9,12].

To determine whether Mtb Pck can bind one or two Mn^2+^ cations in the active site, we titrated the apoenzyme with increasing amounts of Mn^2+^ and detected cation binding by EPR spectroscopy. Calculations revealed that Mtb Pck binds Mn^2+^ with dissociation constant K_D_ = 6.25 ± 3.25 μM and a stoichiometry of binding m = 1.14 ± 0.06, which indicates relatively strong interaction of one Mn^2+^ ion with one protein molecule. These data support the results from our X-ray structure, which indicate that only one Mn^2+^ ion is coordinated in an Mtb Pck metal binding site.

### The Phe triad in the nucleotide binding site is crucial for Pck activity

**Fig 5:**
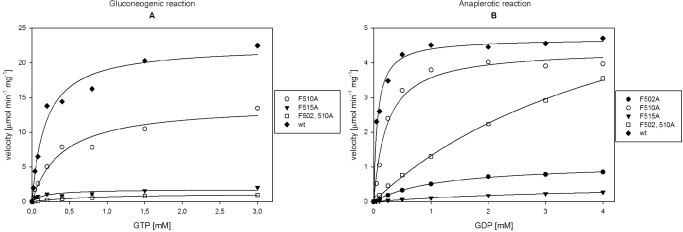
Activities of Pck mutants. (A) Dependence of the gluconeogenic reaction velocities of Pck mutants on GTP concentration. (B) Dependence of the anaplerotic reaction velocities of Pck mutants on GDP. The assays were performed as described in Materials and Methods. The concentrations of individual components were as follows: 2 mM PEP and 2 U/ml MDH for the anaplerotic reaction; 0.3 mM OAA, 10 U/ml LDH, and 3 U/ml PK for the gluconeogenic reaction.

We next examined whether all three Phe residues present in the GTP binding site are critical for the correct binding of GDP and GTP in Mtb Pck and the corresponding enzymatic activities. Mtb Pck mutants with single amino acid substitutions (F502A, F510A, F515A) or double amino acid substitutions (F502A-F510A, F502A-F515A, F510A-F515A) and a triple-mutated variant (F502A-F510A-F515A) were prepared, and purified proteins were tested for catalysis of the gluconeogenic and anaplerotic reactions. Single mutation of Phe in position 502 or 515 almost completely abolished production of PEP or OAA (Fig. 5). These mutations led to increase of K_m_ values by two orders of magnitude for binding of GDP and decrease of maximal velocity that resulted in significant loss of Pck anaplerotic activity (Table 4). Mutation of Phe residues decreased the initial reaction velocities in the gluconeogenic direction compared to wt Pck. The double mutant F502, 510A retained very low gluconeogenic and anaplerotic activities. Mutants F502, 515A, F510, 515A and triple mutant displayed negligible activities. This is in a good agreement with the X-ray structure, which shows that Phe515 forms the highest number of interactions with the guanine base and thus is crucial for stabilization of GDP/GTP in the binding site.

**Table 4:** Kinetic constants for gluconeogenic and anaplerotic reactions catalyzed by wt and F502A; F510A; F515A; F502, 510A; F510, 515A;F502, 515A Pck mutants.

**Gluconeogenic reaction** ^**a**^			
	**V**_**max**_	**K**_**m**_	**V**_**max**_/**K**_**m**_
	**(μmol·min^−1^·mg^−1^)**	**(μM)**	
wt	22.45 ± 1.09	180.8 ± 33.7	0.12
F502A	n.d.	n.d.	
F510A	14.02 ± 1.03	394.4 ± 91.6	0.04
F515A	1.78 ± 0.20	140.4 ± 0.1	0.01
F502, 510A	1.20 ± 0.06	770.1 ± 97.6	0.002
F510, 515A	n.d.	n.d.	
F502, 515A	n.d.	n.d.	
**Anaplerotic reaction** ^**b**^			
	**V**_**max**_	**K**_**m**_	**V**_**max**_/**K**_**m**_
	**(μmol·min^−1^·mg^−1^)**	**(μM)**	
wt	4.69 ± 0.01	66.6 ± 8.0	0.07
F502A	1.11 ± 0.02	1177.2 ± 58.3	9.4·10^–4^
F510A	4.39 ± 0.15	213.7 ± 35.6	0.02
F515A	0.54 ± 0.03	4081.9 ± 422.3	1.3·10^–4^
F502, 510A	7.77 ± 0.46	4877.8 ± 461.4	1.6·10^–3^
F510, 515A	n.d.	n.d.	
F502, 515A	n.d.	n.d.	

^*a*^For gluconeogenic reaction: 100 mM HEPES-NaOH, pH 7.2, 0.3 mM OAA, 2 mM MgCl_2_, 0.2 mM MnCl_2_, 10 mM DTT, 10 U/ml LDH, 3 U/ml PK and 0.2 mM NADH was used.

^*b*^For anaplerotic reaction: 100 mM HEPES-NaOH, pH 7.2, 100 mM KHCO_3_, 37 mM DTT, 2 mM PEP, 2 mM MgCl_2_, 0.1 mM MnCl_2_, 2 U/ml MDH and 0.25 mM NADH was used.

n.d.: Kinetic constants cannot be calculated.

The kinetic measurements were supplemented with thermodynamic analysis of GDP/GTP binding to wt and mutated proteins using isothermal titration calorimetry. The dissociation constants (K_d_) for the interaction between wt Pck and GDP and GTP were 9.3 μM and 7.3 μM, respectively, indicating slightly better affinity of GTP to the Pck binding cleft. The dissociation constants of binding of GDP and GTP to most of the mutants were below the detection limit. The K_d_ value for GDP binding (78 μM) was possible to obtain only for the F510A mutant.

To determine the contribution of each Phe residue to GDP binding, we calculated their energy contribution (ΔΔG'_int_) using a “virtual alanine scan” [37,38]. Energy contributions of individual Phe residues to GDP binding (ΔΔG'_int_) were calculated as the difference between the wt and mutant GDP binding energies (ΔG'_int_). The studied complexes were described by a quantum mechanics (QM) coupled with semiempirical QM (SQM) and molecular mechanics (MM) in hybrid QM/SQM and QM/MM methodologies. The QM region included GDP and mutated residues Phe502, Phe510, and Phe515. The QM/SQM methodology enabled us to use more reliable solvent model. On the other side, the QM/SQM method only allows closed shell calculations. Consequently, we decided to study two complexes. The ternary complex of Pck-GDP-Mn^2+^ (the PDB code 4R43) was only studied by the QM/MM method, while the binary complex of Pck-GDP (the PDB code 4RCG) was analyzed by both the QM/SQM and QM/MM methods for comparison. It should be mentioned here that the conformation and position of GDP and the mutated residues are the same in both crystal structures.(S1 Fig.)

**Fig 6:**
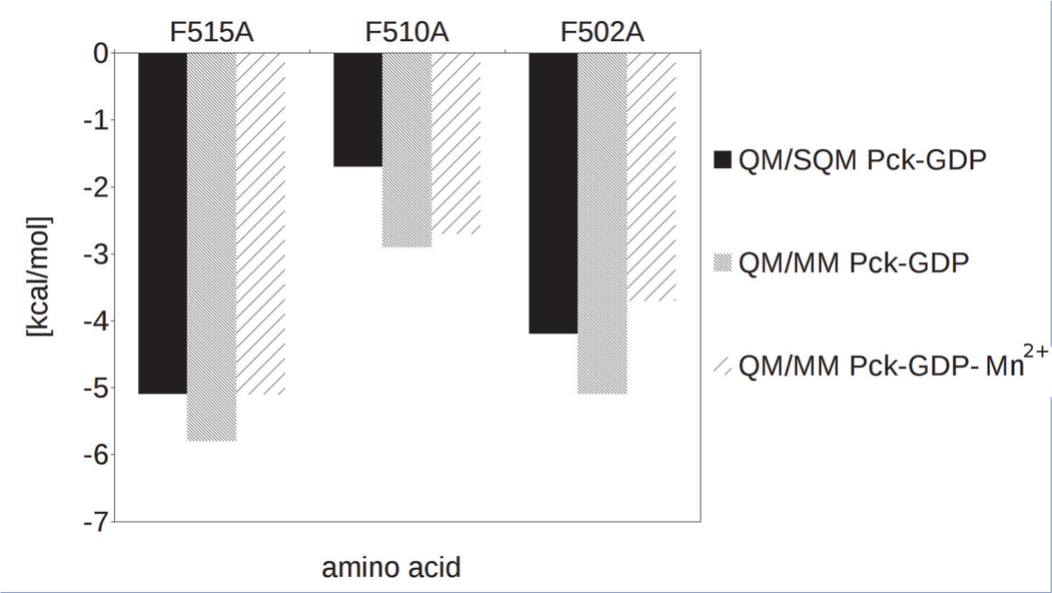
Contribution of Phe residues to GDP binding. The energy contribution (ΔΔG'_int_) of single amino acids to binding of GDP obtained from the “virtual alanine scan.”

Fig. 6 shows the contribution of individual Phe residues to the GDP binding. Both QM/SQM and QM/MM methods as well as both crystal structures give consistent results. Mutation of Phe515 resulted in a loss of binding energy of 5.3 kcal/mol in average, mutation of Phe502 decreased the binding energy by 4.3 kcal/mol in average, and mutation of Phe510 had only moderate influence on the loss of GDP affinity to Mtb Pck (2.4 kcal/mol in average). These results confirmed the key roles of Phe515 and Phe502 for correct positioning and binding of GDP in Pck and for the enzymatic activity.

The dramatic loss of the catalytic activities of the mutated proteins is very likely due to distortion of the GDP/GTP binding pocket. To investigate the influence of Phe mutations on secondary Pck structures, we collected far-UV CD spectra of wt and Phe Pck mutants (S2 Fig.). The greatest secondary structure changes were detected for double mutants F502, 515A and F510, 515A. The total amount of beta-sheets was suppressed in favor of alpha-helices in these mutants (Table 5). These results correspond well with the data obtained from enzyme kinetics and support the finding that Phe515 and Phe502 are important for the proper conformation of the binding site for GDP/GTP in Mtb Pck.

**Table 5:** Circular dichroism analysis: Distribution of secondary structure motifs in wild-type Pck and Pck Phe mutants (F502A; F510A; F515A; F502, 510A; F510, 515A; F502, 510A; F502, 510, 515A).

	α-Helix	β-sheet	β-Turn	Rndm. Coil
**wt-Pck**	24%	24%	17%	35%
**F502A-Pck**	24%	24%	18%	34%
**F510A-Pck**	21%	28%	17%	34%
**F515A-Pck**	19%	28%	17%	36%
**F502, 515A**	52%	11%	16%	21%
**F510, 515A**	50%	12%	17%	20%
**F502, 510A**	22%	26%	18%	34%
**F502, 510, 515A**	26%	25%	18%	32%

## Discussion

Phosphoenolpyruvate carboxykinase plays a pivotal role in central metabolism during fast and slow growth of Mtb [49]. It can catalyze both anaplerotic and gluconeogenic reactions, depending on the reaction conditions [16], and thus can serve as a regulator of the PEP-pyruvate-OAA node *in vivo*. This node distributes the carbon flow within the central carbon metabolism and regulates the distribution of carbon flux among catabolism, anabolism, and the cell energy supply. Regulation of Pck can contribute significantly to the control of Mtb metabolism. Pck and other enzymes participating in catalytic reactions within this node (e.g., pyruvate carboxylase and malic enzyme) are potential targets for drug development. Here, we investigated the regulation of Pck-catalyzed gluconeogenic and anaplerotic reactions by different metabolically relevant divalent cations and performed structural analysis of nucleotide binding site, which has been predicted to be suitable for targeting of small molecular inhibitors [9].

We found that Mtb Pck is present in solution exclusively as a monomer. Neither GDP nor PEP had any effect on the monomeric state of the protein. In contrast, ATP-dependent Pcks typically are multimeric and are subject to allosteric regulation. For example, *Trypanosoma cruzi* Pck is a dimer [18,50], *Saccharomyces cerevisiae* Pck is a tetramer [10,19], and *Urochloa panicoides* Pck is composed of ten units [20,51].

One of the limiting factors controlling Pck-catalyzed reactions is the presence of divalent cations. Both ATP- and GTP-dependent Pcks usually require two cations, which play slightly different roles, for catalytic turnover. In GTP-dependent Pcks, OAA binds to the enzyme and directly coordinates Mn^2+^ in a *cis*-planar arrangement; the second cation, usually Mg^2+^, interacts with GTP bound in a unique site, allowing formation of a catalytic complex [11]. No Mg^2+^ was found in the crystal structures of Mtb Pck-GDP and Pck-GDP-Mn^2+^. Magnesium was not present also in complexes of human Pck with PEP [9], but Mn^2+^ formed a bridge with the terminal oxygen of PEP’s phosphate moiety in the active site. Our biochemical data indicate that Mn^2+^ alone is weak activator of the gluconeogenic reaction catalyzed by Mtb Pck and requires presence of Mg^2+^. The Mtb Pck anaplerotic reaction was activated by Mn^2+^ alone quite efficiently and Mg^2+^ presence even increased dephosphorylation of PEP in this reaction. Comparison of Mtb Pck-GDP and Pck-GDP-Mn^2^ complex structures indicated that binding of Mn^2+^ does not influence positioning of GDP in the active site during anaplerotic reactions. The kinetic data indicated important role of .Fe^2+^ for regulation of Pck catalysis. Activation of Mtb Pck anaplerotic reaction by cations required reducing conditions. Iron activated the Pck anaplerotic reaction and inhibited the gluconeogenic reaction in the presence of Mn^2+^ and Mg^2+^. The importance of iron for growth and survival of Mtb in the host has been shown previously [52]. Elementary analysis of single cations from macrophages infected with Mtb indicated that the concentration of Mn^2+^ does not change over time, in contrast to the Fe^2+^ concentration, which increases by one order of magnitude within 24 h, reaching a concentration of 2680 μM, according to measurements using a hard X-ray microprobe with suboptical resolution [53]. Therefore, it is probable that Fe^2+^ contributes to activation of anaplerotic Pck activity in Mtb surviving in macrophages. Previous work supports the activation of rat liver Pck by Fe^2+^ cations but also reported the necessity of proteins called ferroactivators [54–59] and reduced conditions to protect Pck against deactivation. The cellular ferroactivator was later identified as glutathione peroxidase [60], which significantly increased and prolonged rat Pck activity in the presence of Fe^2+^. Latent Mtb survives in lung granulomas, and a typical characteristic feature of this stage is lack of oxygen, which induces numerous changes in Mtb metabolism [61]. During this stage, the NADH/NAD^+^ ratio increases, and because the terminal electron acceptor is reduced, the pH is slightly acidic in slowly growing Mtb [19,62]. These conditions are also associated with the anaplerotic reaction direction [16–19] and favor activation of Pck by Fe^2+^. Recently, we identified mycobacterial alkyl hydroperoxide reductase C (AhpC, peroxiredoxin 1) as one of the cellular proteins interacting with Mtb Pck [16], and we speculate that AhpC might serve as a ferroactivator of Mtb Pck. AhpC is highly expressed during hypoxic conditions [63], which favors its involvement in Pck protection by interaction with thiols.

Comparison of the crystal structures of Mtb and human cytosolic Pcks revealed a high degree of similarity in the architecture of conserved and functionally specific regions. The GDP pocket is highly specific for a guanosine nucleotide (GDP or GTP), and any rearrangement of its walls by replacement of one of three conserved Phe residues leads to a decrease in or loss of the enzyme’s nucleotide binding properties. The most important contributor to GDP binding is Phe515, which interacts with the guanine ring by π-stacking interaction. Phe515 and Phe502 side chains form a sandwich with GDP base positioned between them. A similar arrangement occurs in rat Pck complexed with GDP [64]. The structure of human cytosolic Pck in complex with GDP is not available, but similar interactions are formed in the human Pck-GTP complex between the corresponding Phe residues and guanine ring of GTP [9]. All three Phe residues have strategic locations within the Pck structure and are crucial for correct positioning of the nucleotide substrate.

Taken together, our results provide evidence that Mtb Pck has different requirements for activation of gluconeogenic and anaplerotic reactions by divalent cations. For effective catalysis in the gluconeogenic direction Mtb Pck requires Mn^2+^ and Mg^2+^ but low micromolar concentrations of Fe^2+^ inhibit this reaction. In contrast, the anaplerotic fixation of carbon dioxide and biosynthesis of oxaloacetate by Mtb Pck can be activated quite effectively by Mn^2+^ and Fe^2+^ alone and simultaneous presence of both cations contributes to more efficient activation of this reaction. Our results show that in addition to reducing conditions, which favor the anaplerotic oxaloacetate synthesis, the composition and concentration of inorganic ions modulate the activity and specificity of Pck and consequently the carbon flow in Mtb. Our structural studies confirmed that Mtb and human Pck are highly similar, which suggests that development of selective Mtb Pck inhibitors targeted against the GTP/GDP binding site will likely be problematic.

**Authors comment:** During the revision of this manuscript, the crystal structures of Mtb apo-Pck and Mtb Pck complex with oxalacetate-Mn^2+^ were deposited by another laboratory in PDB Data Bank (PDB Codes: 4WIE and 4WIU, respectively).

## Supporting Information

S1 Fig Alignment of X-ray Mtb Pck-GDP [PDB code 4RCG] (yellow) and Mtb Pck-GDP-Mn^2+^ [PDB code 4R43] (blue) structures. (TIF)

S2 Fig Far UV CD spectra. wt Pck (black) and Pck Phe mutants (F502A (dark red); F510A (yellow); F515A (apple green); F502, 510A (grass green); F510, 515A (red); F502, 510A (blue); F502, 510, 515A (dotted).
(TIF)
